# Supplementary material for: Cell-morphodynamic phenotype classification with application to cancer metastasis using cell magnetorotation and machine-learning
Source: PLoS One. 2021 Nov 17;16(11):e0259462. doi: 10.1371/journal.pone.0259462 (PMC8598033; doi:10.1371/journal.pone.0259462)
Supplement: S1 File — This document contains detailed description of the dynamic morphology method, both experimental and computational, as well as cell viability controls. (PDF) [file pone.0259462.s001.pdf]

# Cell-morphodynamic phenotype classification with application to cancer metastasis using cell magnetorotation and machine-learning

Remy Elbez[1‡], Jeff Folz[2‡], Alan McLean[3], Hernan Roca[4], Joseph M. Labuz[5], Kenneth J. Pienta[6], Shuichi Takayama[7], Raoul Kopelman[1,2,3*]

**1** Applied Physics Program, University of Michigan, Ann Arbor, Michigan, United States of America
**2** Biophysics Program, University of Michigan, Ann Arbor, Michigan, United States of America
**3** Department of Chemistry, University of Michigan, Ann Arbor, Michigan, United States of America
**4** Department of Urology, University of Michigan School of Medicine, Ann Arbor, Michigan, United States of America
**5** Department of Biomedical Engineering, University of Michigan, Ann Arbor, Michigan, United States of America
**6** The James Buchanan Brady Urological Institute, Department of Urology, Johns Hopkins Hospital, Baltimore, Maryland, United States of America
**7** Department of Biomedical Engineering, Georgia Institute of Technology, Atlanta, Georgia, United States of America

‡These authors contributed equally to this work.
* Corresponding Author: kopelman@umich.edu

# Materials and Methods

## Loading of MNPs

Inductively coupled plasma mass spectrometry (ICP-MS) was used to determine the loading efficiency and average iron nanoparticle concentration per cell utilizing the MDA-MB-231 cell line. ICP-MS was performed on a Perkin-Elmer Nexion 2000 using Fe concentrations of 10, 20, 40, 70, and 100 ppb as standards run under the Helium KED mode.

To prepare samples for ICP-MS, 2 sets of 4 cell plates, each with 500,000 cells, were made in 2 mL of DMEM (Dulbecco's modified eagle media) media. After 24 hours, 70 $\mu$L of 0.2 mg/mL MNP solution (total 0.014 mg/plate) was each added to one set of cell plates. The second set of cell plates with no Fe NP was used as a control. After an additional 24 hour incubation, the media in all plates were aspirated. 1 mL of trypsin was added to each cell plate, cells were incubated for 10 minutes to produce full cellular detachment from the plate and counted on a hemocytometer device (average 575,900 total cells counted), and re-suspended in 1 mL of Milli Q water. Samples were diluted by 1/333 (2 plates per set) and 1/1000 (2 plates per set) to allow the Fe to be within detectable limits for the ICP-MS. Utilizing the ICP calibration curve, iron mass (ppb) was determined for each sample, and undiluted iron concentration was calculated by multiplying the ppb signal by the dilution factor. Next, Fe undiluted concentration was averaged within a set to determine percent uptake (undiluted uptake mass/input Fe NP mass) and divided by the total number of cells to determine NP uptake per cell

(pg/cell). Error (±) represents the standard deviation of the 4 samples values in the set. The control sample (no Fe NP added) did not have a detectable Fe instrument signal under either dilution condition, indicating that the Fe detected in the ICP-MS was purely from the Fe MNP.

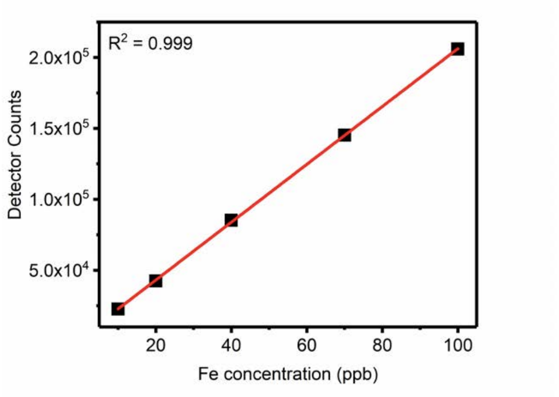


| Percent Uptake of Fe NP | Mass of Iron NP/Cell (pg/cell) |
|---|---|
| 77.6% **± 10.0%** | 18.9 ± 2.5 |

**S1 Fig. Analysis of Magnetic Nanoparticle Uptake** A: ICP Calibration curve used to determine Fe concentration in ICP-MS sample solutions. B: Percent uptake of Fe NP and Mass of Iron NP/Cell (pg/cell) for MDA-MB-231 cells with 7$\mu$g/mL Fe NP incubation.

## Experimental Set-up and Image Acquisition

S2 Fig below shows a cartoon schematic of the experimental set-up. Prior to loading, the microfluidic device is hot glued to a Petri dish. Once the cells have been loaded, the dish is placed on a motorized microscope stage. The Petri dish is then filled with water, which acts as a warm bath to keep the cells in the microfluidic device at 37°C. A thermometer reads the temperature of the microfluidic device's inlet port in real time. Both the Petri dish cap and the motorized stage are equipped with strip heaters, which allow us to actively monitor and maintain temperature. The microfluidic device, Petri dish, and thermometer cable are all covered by a transparent box that ensures that the humidity of the sample is maintained.

Sitting on this box are four solenoids with iron cores. The current running through each solenoid comes from two function generators (Agilent 33220A) outputting sinusoidal currents with a frequency of 15Hz. The phase of these two wave currents is offset by 90°. Both currents pass through an amplifier (Europower EP4000) before going directly to the solenoids themselves. This set-up provides, at the position of the microfluidic device, an oscillating magnetic field with an amplitude of 1mT.

Acquisition of cell images is a fully automated process. Once the microfluidic device is appropriately positioned on stage, we look for a position in the microfluidic device where a high percentage of the microwells contain single cells in the field of view. From 10 to 16 similar areas are then found, and the x, y, and z coordinates of these positions recorded so that the motorized stage can cycle between them automatically. When no images are being taken, a shutter blocks incident light from the microfluidic device to reduce phototoxicity. When images are being taken, the shutter opens for 700ms, allowing us to capture an image of the cells. At each position, an image is taken once every minute for an hour, though we note that our refined decision functions required data from only a single time point.

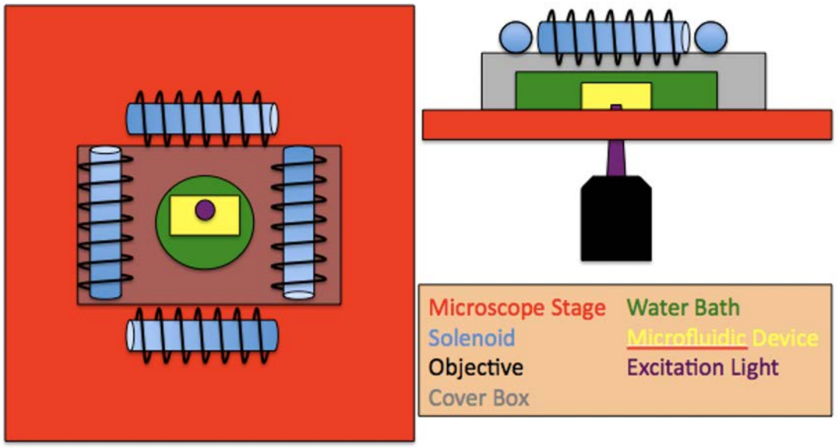


**S2 Fig. A cartoon schematic of the experimental set-up.** On the left, we have a top-down view of the microscope stage while the image on the right shows a cross section. All components sit on a motorized fluorescence microscope stage. The microfluidic device and petri dish (water bath) sit within a cover box for humidity control. The four solenoids sit on this box.

## Viability Test

Cells were stained with Propidium Iodide at times 0 and 60 mins, so as to assess cell viability and cell survival rate during the experiment. In the control test, cells were kept in an incubator after loading in the single-cell trapping device.

S3A Fig shows that the magnetization and rotation of cells has very little effect on cell viability, even over the course of an hour. Figure S3b-e shows the corresponding cell images for the viability assay.

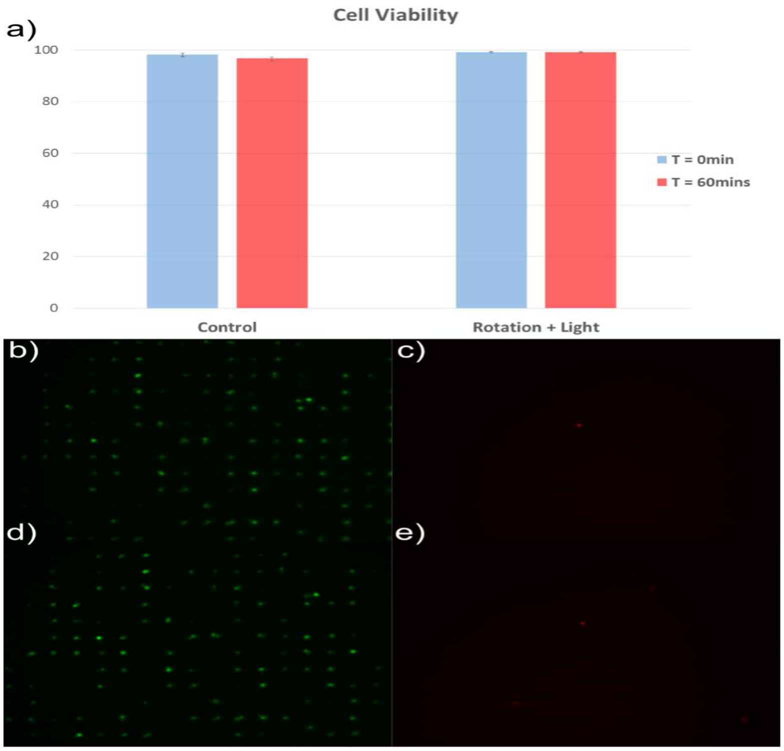

**S3 Fig. Analysis of magnetorotation's effect on cell viability.** A: Live/Dead cell assay for rotated cells exposed to fluorescent light for 60 mins (n=10 samples for each group, N = 1320 cells for control, N=1560 cells for rotated cells). Propidium Iodide (PI) was used to indicate cell death. Blue columns indicate overall cell viability at T=0 mins, after cell loading into the device, and red columns indicate cell survival rate after 60 mins (p-value = 0.125 at a 0.05 significance level). B: GFP expression of MDA-MB-231 cells at T=0 min, C: PI expression at T=0 mins, D: GFP expression of MDA-MB-231 cells at T=60 mins. E: PI expression at T=60 mins. Note: all image colors are pseudocolors.

## Image Processing

After images are gathered, individual cells are cropped to allow CellProfiler to quantify information about each given cell. Here, an exemplary images of MDA-MB-231 cells are provided in the first and third rows, while the morphological delineation found by CellProfiler is given in the second and fourth rows.

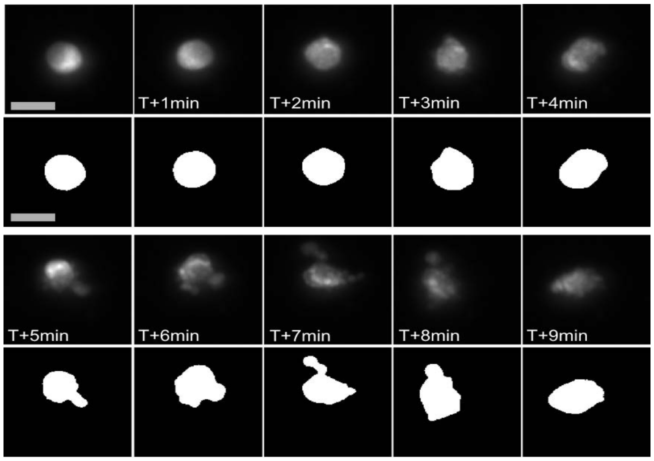


**S4 Fig. Delineation by CellProfiler of a single cell: comparing real cell images to what CellProfiler detects.** Scale bar represents 20$\mu$m. The one cell depicted here is representative of the cells tested. Initially, this cell is rounded, but as time passes it begins to explore it shapes space and reveals itself to be of a motile phenotype. Using our approach, cells that would be difficult to impossible to classify by eye are easily sorted by the computer.

## Feature Generation - Geometric Features

After the cell object in each image has been delineated, we use CellProfiler to measure a variety of parameters in each cell image, which can be broadly broken down into three categories: geometric features, radial features, and texture related features. The geometric features are the easiest to interpret, and include things such as cell area, form factor, orientation, Euler number, extent, eccentricity, axes lengths, and compactness. A complete list with appropriate definitions is found below in S1 Table. Measurements refer or pertain to the cell object.

**S1 Table. Geometric features measured by CellProfiler.** Measurements refer or pertain to the cell object.

| Feature | Definition |
|---|---|
| Area | Total number of pixels inside the cell |
| Center_X | X coordinate of cell center |
| Center_Y | Y coordinate of cell center |
| Eccentricity | Using an ellipse that has the same second moments as the object, eccentricity is the ratio of two distances: 1) the distance from the ellipse's center to a focus, and 2) the distance from that focus to a vertex |
| Euler Number | Number of objects in the region of interest minus the number of holes in those objects |
| Extent | Proportion of pixels in the cell relative to the entire image |
| Form Factor | $Form\ Factor = \frac{4\pi(Area)}{Perimeter^2}$ |
| Maximum/Minimum Feret Diameter | The Feret diameter is the distance between two parallel line drawn on each side of the object. By rotating these lines, we get a set of diameters. |
| Maximum Radius | Largest distance from object center to one of its edge |
| Mean Radius | Average distance from object center to one of its edge |
| Median Radius | Median distance from object center to its edges |
| Major Axis Length | Major axis length of the ellipse that the same second moments as the object |
| Minor Axis Length | Minor axis length of the ellipse that the same second moments as the object |
| Orientation | Angle between the x-axis and the major axis length of the object |
| Perimeter | Total number of pixels constituting the border of the cell |
| Solidity | Variance of the radial pixels of an object divided by the area |

## Feature Generation - Radial Features

The radial features, better known as Zernike moments, are calculated by superimposing a unit disk onto the object of interest, with the unit disk sharing the object's center. All of the coordinates inside the object are converted to polar coordinates, with any coordinate outside of the unit disk (r=1) discarded. The largest disc fitting inside the object is used to calculate the Zernike moments, which are defined below with m and n being two integers and I(x,y) being the intensity of the pixel at the (x,y) coordinate. For the cases where n subtracted from m is even, we have:

$$A_{\mathrm{mn}} = \frac{m+1}{n} \int_x \int_y I(x,y)[Z_{\mathrm{mn}}(x,y)]dxdy \tag{1}$$

where

$$Z_{mn}(x,y) = R_{\mathrm{mn}}(r)e^{jn\theta} \tag{2}$$

and

$$R_{mn} = \sum_{k=0}^{\frac{n-m}{2}} \frac{-1^k(n-k)!}{k!(\frac{m+n}{2}-k)!(\frac{m-n}{2}-k)!} r^{n-2k} \tag{3}$$

If n subtracted from m is odd, the polynomial Rmn becomes 0. In this work, only the first 10 Zernike moments were measured. A table listing the selected values for 'm' and 'n' is found below.

**S2 Table. A list of values taken by the parameters 'm' and 'n' during the calculation of Zernike moments.**

| m | n |
|---|---|
| 0 | 0 |
| 1 | 1 |
| 2 | 0,2 |
| 3 | 1,3 |
| 4 | 0,2,4 |
| 5 | 1,3,5 |
| 6 | 0,2,4,6 |
| 7 | 1,3,5,7 |
| 8 | 0,2,4,6,8 |
| 9 | 1,3,5,7,9 |

To measure radial distributions, the region of interest is split into n concentric circular bins (8 bins were used in this analysis), the center of each bin being the center of the object. FractAtD is the fraction of total stain of the object within a given bin. MeanFrac is the mean fractional intensity at a given radius, and finally, RadialCV is the coefficient of variation of intensity within a ring.

## Feature Generation - Texture Features

The final set of features we measured pertain to texture, and are commonly known as Haralick features. To understand how these features are calculated, we must first introduce the gray-level co-occurrence matrix, G. This matrix is a square matrix of dimension N, where N is the number of values in a grayscale image. Each element G(I,j) is defined as the probability that a pixel of value I is adjacent to a pixel of value j. Pixels in diagonal directions are also considered to be adjacent pixels.

$$G = \begin{pmatrix} p(1,1) & p_(1,2) & \cdots & p_(1,N) \\ p_(2,1) & p_(2,2) & \cdots & p_(2,N) \\ \vdots & \vdots & \ddots & \vdots \\ p_(N,1) & p_(N,2) & \cdots & p_(N,N) \end{pmatrix} \tag{4}$$

Once the coefficients of the co-occurrence matrix are known, a number of additional features may be calculated.

*Angular Second Moment:*

$$\sum_i \sum_j p(i,j)^2 \tag{5}$$

*Contrast:*

$$\sum_{n=0}^{N-1} n^2 \sum_{i=1}^{N} \sum_{j=1}^{N} p(i,j), |i-j| = n \tag{6}$$

*Correlation:*

$$\frac{\sum_i \sum_j p(i,j) - \mu_x \mu_y}{\sigma_x \sigma y} \tag{7}$$

where $\mu$ and$\sigma$ represent the mean and standard deviation of $\mathrm{p_x}$ and $\mathrm{p_y}$, the partial probability functions.

*Sum of Squares Variance:*

$$\sum_i \sum_j (i-\mu)^2 p(i,j) \tag{8}$$

*Inverse difference moment:*

$$\sum_i \sum_j \frac{1}{1+(i-j)^2} p(i,j) \tag{9}$$

*Sum Average:*

$$\sum_{i=2}^{2N} i p_{x+y}(i) \tag{10}$$

Where x and y are the row and column of an entry in the co-occurrence matrix, and $\mathrm{p_{x+y}}$(i) is the probability of the co-occurrence matrix coordinates summing to x+y.

*Sum Variance:*

$$\sum_{i=2}^{2N} (i-f_8)^2 p_{x+y}(i) \tag{11}$$

where $\mathrm{f_8}$ is the sum entropy:

$$f_8 = -\sum_{i=2}^{2N} p_{x+y}(i) log(p_{x+y}(i)) \tag{12}$$

*Difference Variance:*

$$\sum_{i=0}^{N-1} p_{x-y}(i) log(p_{x-y}(i)) \tag{13}$$

*Information Measure of Correlation 1:*

$$\frac{HXY - HXY_1}{max(HX, HY)} \tag{14}$$

*Information Measure of Correlation 2:*

$$\sqrt{1 - e^{HXY_2 - HXY}} \tag{15}$$

where HX and HY are the entropies of $\mathrm{p_x}$ and $\mathrm{p_y}$, and

$$HXY = -\sum_{i}\sum_{j} p(i,j) log(p(i,j)) \tag{16}$$

$$HXY_1 = -\sum_{i}\sum_{j} p(i,j) log[p_x(i) p_y(j)] \tag{17}$$

$$HXY_2 = -\sum_{i}\sum_{j} p_x(i) p_y(j) log[p_x(i) p_y(j)] \tag{18}$$
