# Supplementary material for: Cell-morphodynamic phenotype classification with application to cancer metastasis using cell magnetorotation and machine-learning
Source: PLoS One. 2021 Nov 17;16(11):e0259462. doi: 10.1371/journal.pone.0259462 (PMC8598033; doi:10.1371/journal.pone.0259462)
Supplement: Attachment — Submitted filename: Response to Reviewers.docx [file pone.0259462.s002.docx]

***Response to Reviewers***

***1. The authors have presented their data primarily on the two breast cancer cell lines with some insight into a prostate cancer cell line. Can a healthy (non-cancerous) cell line be tested as a control to test their hypothesis?***

We thank the reviewer for this comment, which is to verify that our technique can successfully resolve healthy and cancerous phenotypes. This task would necessitate the creation of a stable, GFP-expressing healthy breast line. Unfortunately, we have been unable to find healthy (non-cancerous) GFP-expressing epithelial or mesenchymal breast lines, such as within human mammary epithelial cells (HMECs), neither from cell vendors (e.g., ATTC) nor from laboratories within the University of Michigan. This would mean that we would need to create the GFP-expressing cell line within our laboratory from a non-GFP expressed base; unfortunately, while this process is possible through transfection, it will take an extended time, involve substantial costs, and would require control testing (serial passaging, etc.) once the GFP expression is achieved. We thank the reviewer for this insightful comment, and address it by having added several citations comparing cancer and non-cancerous cells. For example, Joshi et al. were able to differentiate triple negative breast cancer from healthy breast cells using an approach that combined machine learning with impedance microcytometry. Using impedance alone enabled successful classification of cancerous vs non-cancerous cells in 86.5% of instances. By including additional features such as phase change and current change, the success rate climbed to 97.3%. In another case, Hasan colleagues utilized time lapse fluorescent microscopy to track cell movements overtime. After extracting features form their images using a level-set algorithm, they were able to classify cancerous from non-cancerous cells using a naïve bayes classifier with 85% accuracy. Thus, we have added the following to the manuscript so as to highlight and make explicit these previous approaches, in contrast to ours:

“While previous studies have been conducted that differentiate cancerous from non-cancerous cells (27, 28), we have extended this kind of analysis so as to compare metastatic with non-metastatic cancer cell types.”

***2. Why were the magnetic nanoparticles used? Was it for growing spheroids? This portion must be clarified***

We thank the reviewer for this comment. To help clarify the role of the MNPs in this study, we have adjusted the introduction so as to include the following information:

“…, *we use magnetic nanoparticles so as to trap, suspend, and rotate cells that are captured in a microfluidic chamber (29). Magnetorotation prevents cells from adhering the microwells and permits exploration of their morphodynamic space.”*

Summarizing, in this study, the MNPs play no role in the formation of spheroids. They are used to prevent cells from adhering to the surface, so they can express their “shape-shifting” ability (see Fig S4 and previous group publication by Elbez. et. al. DOI: [10.1371/journal.pone.0028475](https://doi.org/10.1371/journal.pone.0028475) (ref 29)).

***3. Can the authors comment on what the significance of this method would be on cancers with a late prognosis with no early biomarkers available (pancreatic cancer)?***

We very much thank the reviewer for this comment. As our technique does not rely on specific biomarkers, but instead on the delineation of a cell’s morphology via fluorescence imaging, it may be able to identify pre-cancerous or cancerous morphological phenotypes present in pancreatic cancers. By detecting these phenotypes, our technique may be of tremendous diagnostic value by enabling their early detection. The following quote has thus been added to our discussion section:

“Importantly, early identification of cancerous or pre-cancerous morphodynamic phenotypes could enable early detection of cancers which produce no known biomarkers, such as is the case for pancreatic cancer (50, 51). In future work, it would be beneficial to extend the dynamic morphodynamics platform to demonstrate sensitive differentiation of such cells, which could be of important diagnostic value.”

***4. The following references should be added:***
<https://pubmed.ncbi.nlm.nih.gov/21553120/>
<https://pubmed.ncbi.nlm.nih.gov/33745223/>
<https://pubmed.ncbi.nlm.nih.gov/33151075/>
<https://pubs.acs.org/doi/abs/10.1021/jacs.6b12236>

We thank the reviewer for bringing these literature papers to our attention. These references have now all been added to the main text:

In the introduction:

“It has also been reported that *morphological changes* can be used to identify cells having undergone the EMT *(10,* [https://pubmed.ncbi.nlm.nih.gov/21553120/*)*](https://pubmed.ncbi.nlm.nih.gov/21553120/))*.”*

In the discussion:

“While subtle differences in nanoparticle formulations have been shown to effect cell responses to therapy (<https://pubmed.ncbi.nlm.nih.gov/33745223/>, <https://pubmed.ncbi.nlm.nih.gov/33151075/>), our technique has been demonstrated to have minimal impact on cell viability (Fig S2).”

“Importantly, we can track behaviors and morphological changes that are intrinsic to a *label free*, floating or circulating, cell, without relying on any biomarker, antibody, or on any *a priori* knowledge of the genotype, which are often required for other CTC-capturing techniques (<https://pubs.acs.org/doi/abs/10.1021/jacs.6b12236>, 38).”

In summary, we thank the reviewer for his many helpful comments, apologize for not being clear re the MNP role, and believe that we have now adjusted the manuscript so as to benefit from these comments.
